# Cloning, Purification, and Characterization of a β-Carbonic Anhydrase from *Malassezia restricta*, an Opportunistic Pathogen Involved in Dandruff and Seborrheic Dermatitis

**DOI:** 10.3390/ijms20102447

**Published:** 2019-05-17

**Authors:** Sonia Del Prete, Daniela Vullo, Cynthia Ghobril, Julien Hitce, Cécile Clavaud, Xavier Marat, Clemente Capasso, Claudiu T. Supuran

**Affiliations:** 1 Istituto di Bioscienze e Biorisorse, CNR, Via Pietro Castellino 111, 80131 Napoli, Italy; sonia.delprete@ibbr.cnr.it; 2 Dipartimento Neurofarba, Sezione di Scienze Farmaceutiche e Nutraceutiche, Università degli Studi di Firenze, Via U. Schiff 6, 50019 Sesto Fiorentino, Florence, Italy; daniela.vullo@unifi.it; 3 L’Oréal Research and Innovation, 93601 Aulnay-sous-Bois, France; cynthia.ghobril@rd.loreal.com (C.G.); julien.hitce@rd.loreal.com (J.H.); cecile.clavaud@rd.loreal.com (C.C.); xavier.marat@rd.loreal.com (X.M.)

**Keywords:** carbonic anhydrase, *Malassezia restricta*, cloning, enzyme inhibition, acetazolamide

## Abstract

The cloning, purification, and initial characterization of the β-carbonic anhydrase (CA, EC 4.2.1.1) from the genome of the opportunistic pathogen *Malassezia restricta* (MreCA), which a fungus involved in dandruff and seborrheic dermatitis (SD), is reported. MreCA is a protein consisting of 230 amino acid residues and shows high catalytic activity for the hydration of CO_2_ into bicarbonate and protons, with the following kinetic parameters: k_cat_ of 1.06 × 10^6^ s^−1^ and k_cat_/K_M_ of 1.07 × 10^8^ M^−1^ s^−1^. It is also sensitive to inhibition by the sulfonamide acetazolamide (K_I_ of 50.7 nM). Phylogenetically, MreCA and other CAs from various *Malassezia* species seem to be on a different branch, distinct from that of other β-CAs found in fungi, such as *Candida* spp., *Saccharomyces cerevisiae*, *Aspergillus fumigatus*, and *Sordaria macrospora*, with only *Cryptococcus neoformans* and *Ustilago maydis* enzymes clustering near MreCA. The further characterization of this enzyme and the identification of inhibitors that may interfere with its life cycle might constitute new strategies for fighting dandruff and SD.

## 1. Introduction

Carbonic anhydrases (CA, EC 4.2.1.1) catalyze the simple but physiologically crucial interconversion of carbon dioxide and water into bicarbonate and protons: CO_2_ + H_2_O ⇌ HCO_3_^−^ + H^+^ [1,2,3,4,5]. These metalloenzymes are indispensable for maintaining the physiological equilibrium of the dissolved CO_2_, H_2_CO_3_, HCO_3_^−^, and CO_3_^2−^, which are metabolites essential for the biosynthesis and energy metabolism of organisms [5,6,7,8,9]. Thus, the survival of a microbe will be compromised by restricting the access of a pathogen to these metabolites. Interference with pH regulation, as well as metabolic pathways connected with the inhibition of CA activity, has begun to be considered for obtaining new anti-infective agents [10,11,12,13,14], in addition to applications of such agents for antitumor therapies [14,15,16,17]—the field in which this approach was validated after more than a decade of strenuous research efforts [6,7,18,19,20,21]. Indeed, the 21st century has been affected by the spread of antibiotic resistance, and consequently, the improvement of the pharmacological arsenal against pathogens is needed [22,23,24,25,26]. Most of the existing antibiotics target a microorganism’s cellular functions, such as the synthesis of proteins, nucleic acids, cell walls, or folate [27,28,29]. Generally, the identification of novel drug targets follows criteria pointing to pathogen survival and absence from the human genome [30,31,32]. Recently, it has been demonstrated that the CA superfamily represents a valuable member of new macromolecules affecting the growth of microorganisms or making them vulnerable to host defense mechanisms [32,33,34]. Targeting CAs from pathogens belonging to various species of bacteria, fungi, and protozoans may lead to anti-infectives with new mechanisms of action different from the clinically used agents [34,35,36,37]. Among the fungal pathogens investigated in detail, *Malassezia globosa* (MgCA) represents a particular case [38,39,40,41], because the β-CA encoded in the genome of this fungus was shown to be a druggable target [41]. Indeed, several effective in vitro MgCA inhibitors belonging to the sulfonamide type were also shown to have significant antifungal effects in vivo, in an animal model of dandruff [42,43,44]. Thus, after considering the significant drug resistance problems with azoles and other antifungals [45,46,47,48,49], MgCA was validated as a possible antifungal target.

Recently, it has been demonstrated that, rather than one particular *Malassezia* sp. being involved, a complex bacterial and fungal equilibrium is involved in dandruff [50,51,52,53,54,55,56]. Another *Malassezia* species, *Malassezia restricta* is also involved in triggering the disequilibrium between the commensals *Cutibacterium acnes* (formerly named *Propionibacterium acnes*) and *Staphylococcus* sp., both of which contribute to dandruff and seborrheic dermatitis symptoms [50,51,52,57]. Furthermore, the genome of this pathogen was recently decoded and was published [57], making it possible to search for potential drug targets and the corresponding agents that may interfere with them. For this reason, we decided to investigate the analogous enzyme from MgCA, which should also be present in the genome of *M. restricta*. here, we report on the cloning, purification, and characterization of the β-CA from the pathogenic fungus, *M. restricta* (MreCA).

## 2. Results and Discussion

### 2.1. MreCA Features

**Figure 1:**
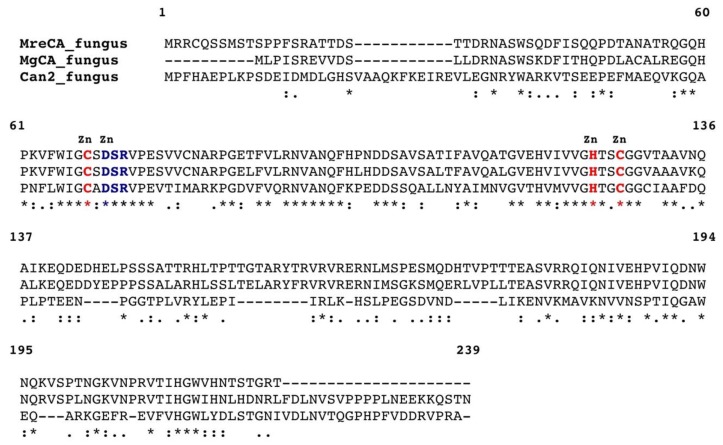
Multiple sequence alignment of selected β-carbonic anhydrase (β-CAs) from three fungal species. The *Cryptococcus neoformans* CA (Can2) numbering system was used. Zinc ligands are indicated in red, and amino acids involved in the enzyme catalytic cycle are indicated in blue. Multiple sequence alignment was performed with the program Clustal W, version 2.1. Legend: *Malassezia restricta* (MreCA), *Malassezia globosa* (MgCA), and *Cryptococcus Neoformans* (Can2). Conserved residues are indicated with an asterisk (*), while (:) and (.) indicate conservative and semi-conservative substitutions, respectively. The sequence accession numbers are reported in Table 1.

The genome of *M. restricta* contains a region of 690 bp encoding for a polypeptide chain of 230 amino acid residues and is homologous to the β-CA identified in the genome of *M. globosa* (MgCA). To show the relevant degree of homology existing between these enzymes, MreCA was aligned with MgCA and *Cryptococcus neoformans* CA (Can2) [58,59], as shown in Figure 1.

**Figure 2:**
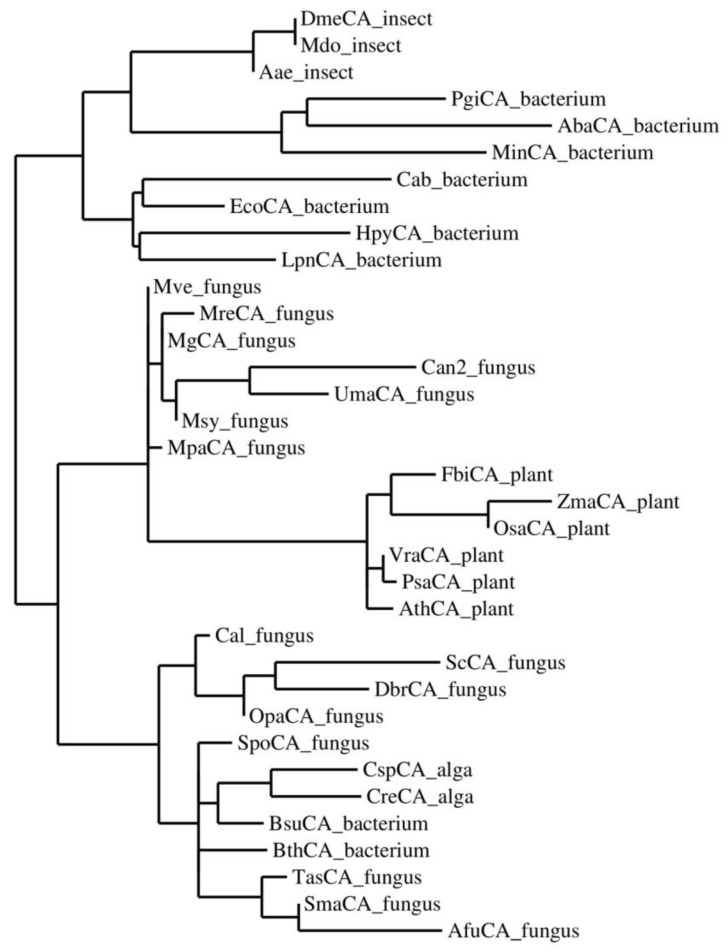
Phylogenetic tree of the β-CAs from selected prokaryotic and eukaryotic species. The tree was constructed using PhyML 3.0. For the acronyms and organism names see Table 1.

MreCA contains all the typical features of β-CAs, including the three residues that are involved in the catalytic mechanism of the enzyme, acting as zinc ligands (two cysteines and one histidine). It also contains amino acid residues, including the catalytic dyad that consists of an aspartate and an arginine, which are involved in the activation of the zinc-coordinated water molecule responsible for nucleophilic attack [38,39,40,41,42,43,44,45,58,59] (Figure 1). To better investigate the relationships between MreCA and the β-CAs identified in other species, such as insects, plants, fungi, algae, and bacteria, the most parsimonious phylogenetic tree has been constructed (Figure 2), which takes into account all the amino acid substitutions that differentiate β-CAs from various organisms indicated in Table 1.

From the dendrogram reported in Figure 2, MreCA appears to be closely related to the β-CAs identified in the genome of the other *Malassezia* species. Intriguingly, the MreCA and MgCA clusters included the β-CA encoded by the genome of the pathogenic fungus *Ustilago maydis*, which is responsible for a plant disease known as common corn smut [60]. It has been reported that the genomes of *M. restricta* and *globosa* encode for many extracellular hydrolases, such as lipases, phospholipases, aspartyl proteases, and acid sphingomyelinases, which are phylogenetically close to those encoded by the *U. maydis* genome [60]. The dendrogram in Figure 2 also shows that the *Malassezia* β-CAs are neighbors to the β-CA from *Cryptococcus neoformans* (Can2). They are clustered distinctly away from the other β-CAs found in the genome of fungi, such as *Candida albicans*, *Saccharomyces cerevisiae*, *Dekkera bruxellensis*, *Ogataea parapolymorpha*, *Aspergillus fumigatus*, *Sordaria macrospora*, *Trichosporon asahii*, and *Schizosaccharomyces pombe*. This result may be the consequence of a gene duplication event indicating an ancestral β-CA gene in these two groups of fungi. Furthermore, *Malassezia* CAs are in the same cluster as the β-CAs from plants (Figure 2).

### 2.2. Expression, Purification, and Protonography

**Figure 3:**
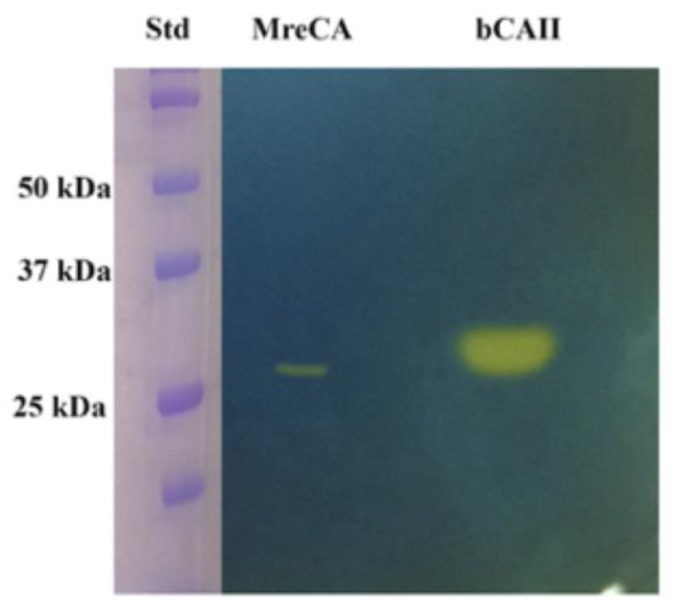
Protonography of MreCA with bovine CA (bCAII) as the standard enzyme.

IPTG (Isopropyl β-D-1-thiogalactopyranoside) induction of *Escherichia coli* BL21 (DE3) cells transformed with the plasmid pET100D-Topo/MreCA resulted in the production of the recombinant MreCA as a fusion protein containing a His-tag tail at its N-terminus. After sonication and centrifugation, most of the CA activity was recovered in the soluble fraction of the *E. coli* cell extract. Using an affinity column (His-select HF (High Flow) nickel affinity gel), MreCA was purified to homogeneity, as shown by the appearance of the SDS-PAGE results (material not intended for publication). Samples of the purified MreCA were loaded onto the gel and subjected to protonography to investigate its hydratase activity via SDS–PAGE. Protonography is a powerful technique, which allows the detection of pH variation on polyacrylamide gel due to the CA-catalyzed conversion of CO_2_ into bicarbonate and protons [61,62]. The production of ions (H^+^) during the CO_2_ hydration reaction can be visualized as a yellow band on the polyacrylamide gel (Figure 3). The developed gel is called a protonogram.

The MreCA protonogram in Figure 3 shows a band corresponding to a monomer with an apparent molecular weight of about 27.0 kDa. The predicted molecular mass of the enzyme fused to the His-tag tail resulting from its amino acid sequence was 27.0 kDa. It is interesting to note that, as was found for other CA classes belonging to prokaryotic and eukaryotic organisms, MreCA was able to correctly refold, generating the active enzyme after removing SDS from the gel before developing the protonogram. Commercial bovine CA was used as a positive control.

### 2.3. Determination of the Kinetic Constants

Using stopped-flow techniques, the kinetic parameters were determined for the purified recombinant MreCA using CO_2_ as a substrate. The data in Table 2 demonstrate that MreCA shows a catalytic activity higher than that of MgCA, with the following kinetic parameters: k_cat_ of 1.06 × 10^6^ s^−1^ and k_cat_/K_M_ of 1.07 × 10^8^ M^−1^ s^−1^. Therefore, when compared with the high-activity human isoform hCA II, it is only slightly less effective as a catalyst for CO_2_ hydration. Furthermore, the activity is highly inhibited by acetazolamide, the clinically used sulfonamide inhibitor, with an inhibition constant of 50.7 nM, as seen in Table 2. Thus, the two *Malassezia* enzymes, MgCA and MreCA, show a net difference in their sensitivity to this sulfonamide, with the first one being quite resistant to the inhibitor and the second one being quite sensitive. It is interesting to stress the fact that β-CAs are missing in the genome of *Cutibacterium acnes* and *Staphylococcus epidermidis*, the two most abundant bacteria found on the human scalp. Thus, the synthesis of new drugs capable of interfering with MreCA and MgCA activity will not influence the catalytic mechanism of the CAs encoded by the scalp microbes, preserving not only the integrity of the human skin, but also avoiding interference with human CAs, because the human genome only encodes CAs belonging to the α-class.

## 3. Materials and Methods

### 3.1. Cloning and Purification of MreCA

The genome of *M. restricta* used in this study to retrieve the amino acid sequence was recently published [57]. The amino acid sequence of *MreCA* is shown in Figure 1. It was back translated into the nucleotide sequence and optimized for codon usage to increase its expression in *E. coli* cells. The synthetic *M. restricta* gene, as obtained from GeneArt Company (Milan, Italy), contained four base-pair sequences (CACC) necessary for directional cloning at the 5′ end of the MreCA DNA gene. The fragment was cloned into the expression vector pET100/D-TOPO (Invitrogen, Carlsbad, CA, USA), creating the plasmid pET100D-Topo/MreCA. Competent *E. coli* BL21 (DE3) codon plus cells (Agilent, Santa Clara, CA, USA) were transformed with the pET100D-Topo/MreCA. The level of expression of the target protein was improved by varying the time and temperature of induction and the concentration of the inducer (IPTG). After growth, the cells were harvested and disrupted by sonication at 4 °C in 20 mM of phosphate buffer, pH 8.0. Following sonication, the sample was centrifuged at 1200× *g* at 4 °C for 30 min. The supernatant was loaded onto a His-select HF nickel affinity column. The MreCA was eluted with 0.02 M phosphate buffer (pH 8.3) containing 250 mM imidazole and 0.5 M NaCl at a flow rate of 1.0 mL/min. Fractions were collected and dialyzed. At this stage of purification, the enzyme was at least 80% pure, and the obtained recovery was of 0.1 mg of the recombinant MreCA per liter of culture. The catalyst showed activity after undergoing the protonography test [61,62] (Figure 3) and via a kinetic, stopped-flow CO_2_ hydrase assay [63] (Table 2).

### 3.2. Protonography

Wells of 12% SDS gel were loaded with MreCA or the commercial bovine CA (Sigma, St. Louis, MO, USA), mixed with Laemmli loading buffer containing SDS (1% final concentration) but without 2-mercaptoethanol. In order to avoid protein denaturation the samples were not boiled. The gel was run at 150 V until the dye front moved off the gel. Following electrophoresis, the 12% SDS gel was subjected to protonography in order to detect the MreCA hydratase activity on the gel as described by Capasso et al. [61,62].

### 3.3. SDS-PAGE, Primary Structure, and Phylogenetic Analysis

Sodium dodecyl sulfate (SDS) polyacrylamide gel electrophoresis (PAGE) was performed using 12% gels as described previously [61,62,64]. Multiple amino acid sequence alignment of the amino acid sequences of the β-CAs from the different species was performed with the program CLUSTAL W, version 2.1 [65]. The phylogenetic tree of β-CAs from the selected prokaryotic and eukaryotic species was constructed by using the program PhyML 3.0 [66].

### 3.4. CA Activity Measurements

An applied photophysics stopped-flow instrument was used for assaying the CA-catalyzed CO_2_ hydration activity [19]. Bromothymol blue (at a concentration of 0.2 mM) was used as an indicator, working at the absorbance maximum of 557 nm, with 10–20 mM TRIS (pH 8.3) as a buffer and 20 mM Na_2_SO_4_ for maintaining the ionic strength (this anion is not inhibitory and has a K_I_ > 200 mM against this enzyme), following the initial rates of the CA-catalyzed CO_2_ hydration reaction for a period of 10–100 s. In order to determine the kinetic parameters and inhibition constants, the CO_2_ concentrations ranged from 1.7 to 17 mM. For each measurement, at least six traces of the initial 5%–10% of the reaction were used for determining the initial velocity, working with 10-fold decreasing inhibitor concentrations ranging from 1 nM to 10–100 µM (depending on the inhibitor potency, but at least 5 points at different inhibitor concentrations were employed for determining the inhibition constants). The uncatalyzed rates were determined in the same manner and then subtracted from the total observed rates. Stock solutions of inhibitor (0.1 mM) were prepared in distilled-deionized water, and dilutions up to 1 nM were done thereafter with the assay buffer. Inhibitor and enzyme solutions were pre-incubated together for 15 min at room temperature before assaying, in order to allow for the formation of the E–I complex. The inhibition constants were obtained by non-linear least-squares methods using the Cheng–Prusoff equation, and represent the mean from at least three different determinations. The human isoforms hCA I, II, and IX were assayed in the same conditions as above except that the working pH was 7.4, using HEPES (4-(2-hydroxyethyl)-1-piperazineethanesulfonic acid) buffer and phenol red as an indicator [63]. All enzymes were recombinant ones produced in our laboratory as described previously [58,59,63,67,68,69,70].

### 3.5. Multiple Sequence Alignment and Phylogenetic Analysis

Multiple alignment of the amino acid sequences of β-CAs identified in the genome of organisms belonging to different groups (bacteria, fungi, algae, insects, and plants) was performed with the program CLUSTAL W, version 2.1 [65]. The phylogenetic tree of β-CAs from the selected prokaryotic and eukaryotic species was constructed using the program PhyML 3.0, which searched for the tree with the highest probability [66].

## 4. Conclusions

We report here on the cloning, purification, and initial characterization of the β-CA (MreCA) from the genome of the pathogenic fungus *M. restricta*, responsible for dandruff, together with other fungi and bacteria. MreCA has a high catalytic activity for the hydration of CO_2_ into bicarbonate and protons, with the following kinetic parameters: k_cat_ of 1.06 × 10^6^ s^−1^ and k_cat_/K_M_ of 1.07 × 10^8^ M^−1^ s^−1^. It is also sensitive to inhibition by the classical sulfonamide inhibitor acetazolamide (K_I_ of 50.7 nM). Phylogenetically, MreCA and other CAs from various *Malassezia* species seem to be on a different branch, distinct from that of other β-CAs found in fungi, such as *Candida* spp., *Saccharomyces cerevisiae*, *Aspergillus fumigatus*, and *Sordaria macrospora*, with only the *Cryptococcus neoformans* and *Ustilago maydis* enzymes being on the same branch as MreCA. As previously reported, the closest species were of plant origin. The further characterization of this enzyme and identification of inhibitors that could interfere with its life cycle might constitute new strategies for fighting dandruff and seborrheic dermatitis.

## Figures and Tables

**Table 1:** The accession numbers of the β-CA sequences used in the phylogenetic analysis. Groups, organism names, and acronyms are reported.

Group	Organism Name	Acronym	Accession Number
**Bacteria**			
	*Porphyromonas gingivalis*	PgiCA_bacterium	YP_001929649.1
	*Acinetobacter baumannii*	AbaCA_bacterium	YP_002326524
	*Myroides injenensis*	MinCA_bacterium	ZP_10784819
	*Methanobacterium thermoautotrophicum*	Cab_bacterium	GI:13786688
	*Helicobacter pylori*	HpyCA_bacterium	BAF34127.1
	*Legionella pneumophila*	LpnCA_bacterium	YP_003619232
	*Escherichia coli*	EcoCa_bacterium	ACI70660
	*Burkholderia thailandensis*	BthCA_bacterium	ZP_02386321
	*Brucella suis*	BsuCA_bacterium	NP_699962.1
**Fungi**			
	*Malassezia globosa*	MgCA_fungus	XP_001730815.1
	*Malassezia pachydermatis*	MpaCA_fungus	XP_017991749.1
	*Malassezia vespertilionis*	Mve_fungus	PKI85431.1
	*Malassezia sympodialis*	Msy_fungus	XP_018739548.1
	*Malassezia restricta*	MreCA_fungus	PRJNA474956
	*Cryptococcus neoformans*	Can2_fungus	GI:219109194
	*Candida albicans*	Cal_fungus	XP_721792.1
	*Saccharomyces cerevisiae*	ScCA_fungus	GAA26059
	*Dekkera bruxellensis*	DbrCA_fungus	EFW97556
	*Ogataea parapolymorpha*	OpaCA_fungus	EFW97556
	*Aspergillus fumigatus*	AfuCA_fungus	XP_751704
	*Sordaria macrospora*	SmaCA_fungus	CAT00781
	*Trichosporon asahii*	TasCA_fungus	EKD04029
	*Schizosaccharomyces pombe*	SpoCA_fungus	CAA21790
	*Ustilago maydis*	UmaCA_fungus	XM_011388340.1
**Algae**			
	*Coccomyxa* sp.	CspCA_alga	AAC33484.1
	*Chlamydomonas reinhardtii*	CreCA_alga	XP_001699151.1
**Insect**			
	*Drosophila melanogaster*	DmeCA_insect	NP_649849
	*Musca domestica*	Mdo_insect	XP_005191496.1
	*Aedes aegypti*	Aae_insect	XP_021707077.1
**Plant**			
	*Vigna radiata*	VraCA_plant	AAD27876
	*Pisum sativum*	PsaCA_plant	AAA33652
	*Flaveria bidentis*	FbiCA_plant	AAA86939.2
	*Arabidopsis thaliana*	AthCA_plant	AAA50156
	*Zea mays*	ZmaCA_plant	NP_001147846.1
	*Oryza sativa*	OsaCA_plant	AAA86943

**Table 2:** Kinetic parameters for the newly obtained enzyme from MreCA, compared with the human hCA II (α-class) and the β-class enzyme from MgCA at 25 °C, pH 8.3 in 20 mM Tris buffer and 20 mM NaClO_4_, for the CO_2_ hydration reaction.

Isozyme	k_cat_ (s^−1^)	K_M_ (mM)	k_cat_/K_M_ (M^−1^ s^−1^)	K_I_ (AAZ) (nM)
hCA II	1.4 × 10^6^	9.3	1.4 × 10^8^	12
MgCA	9.2 × 10^5^	11.1	8.3 × 10^7^	74,000
MreCA *	1.06 × 10^6^	10.1	1.07 × 10^8^	50.7

* The kinetic/inhibition parameters are the mean from 3 different assays. Errors are in the range of 10% of the reported values (material not intended for publication).
